# Characteristics of anatomical landmarks in the mandibular interforaminal region: A cone-beam computed tomography study

**DOI:** 10.4317/medoral.17520

**Published:** 2011-12-06

**Authors:** Fereidoun Parnia, Elnaz Moslehifard, Ali Hafezeqoran, Farhang Mahboub, Haniye Mojaver-Kahnamoui

**Affiliations:** 1 DDS, MSc, Assistant professor, Department of Prosthodontics, School of Dentistry, Tabriz University of Medical Sciences, Tabriz, Iran; 2 DDS, MSc, Assistant professor, Department of Prosthodontics and Dental and Periodontal Research Center, School of Dentistry, Tabriz University of Medical Sciences, Tabriz, Iran; 3 DDS, Private Practice

## Abstract

Objectives: This study was conducted to assess appearance, visibility, location and course of anatomical landmarks in mandibular interforaminal region using cone-beam computed tomography (CBCT). Study design: A total of 96 CBCT examinations was re-evaluated to exploit anatomical landmarks. The examinations used the Promax 3D CBCT unit. A sole examiner carried out all the measurements. Visibilities of the anatomical landmarks were scored using a four-point rating scale. Results: The mandibular foramen, anterior loop, incisive canal and lingual foramen were observed in 100,84,83,49 % of the images, respectively. The mean size, diameter and width of anterior loop, incisive canal and lingual foramen were obtained 3.54± 1.41, 1.47±0.50 and 0.8 ± 0.09mm, respectively. Conclusion: It is not safe to recommend any definite distance mesially from the mental foramen. The diameter of the canals and foramens should be determined on a case-by-case basis to exploit the appropriate location for each individual.

** Key words:**Anatomical landmarks, cone-beam computed tomography, implant surgery, radiographic evaluation, surgical complications.

## Introduction

The mandibular inter-mental area is assumed to be a safe area for implant insertion and is involved in many other surgical procedures. It is essential to understand the anatomy of the region for avoiding injuries to the neurovascular bundles. Provided that the inferior alveolar or mental nerve is damaged during preparation of an osteotomy, sensory dysfunction appears due to nerve damage in the foraminal area. Recently developed all-on-four procedure permits a quick placement of 4 dental implants in the interforaminal area of the lower jaw associated with a fixed bridge during only one appointment. The mental foramen is a strategically important landmark during osteotomy procedures. The inferior alveolar nerve may extend beyond the mental foramen as an intraosseous anterior loop. The location of foramen, as well as, the possibility that an anterior loop of the mental nerve may be present mesial to the mental foramen need to be considered before implant surgery to avoid mental nerve injury. The mandibular canal contains the inferior alveolar nerve and blood vessels and is divided into mental and incisive segments between the roots of premolars. The mental canal deviates toward the mental foramen. However, the incisive canal continues below the incisor teeth where it generally is divided into a plexus of nerve branches until its main trunk is lost (1-3). In this area lingual foramen is also present that is located in the midline, leveled with or superior to the genial tubercles. The foramen has a branch of the incisive artery that anastomosis with the lingual artery (4).

To establish a zone of safety (in millimeters) in implant placement, both the available bone coronal and anterior to the foramen are quite important. Any improper implant insertion anterior to the mental canal can cause oedema of the epineurium. This may spread to the main mental branch, leading to neurosensory disturbances. Some authors have reported the evidences of discomfort, pain, and disturbance of sensation after implant insertion into the inter-mental area (5-7). Rosenquist assumed that implant failure and neurosensory dysfunction were partly due to the large diameter of the mandibular incisive canal. In this report, it was found that incisive bundle causes implant failure, by migrating of soft tissue around the implant, thus preventing osteointegration (8). In addition, sensory disturbances of the mental nerve region may arise after endosseous implants are installed in the mandibular interforaminal region because of damage to anatomical structures in this area (9). For these reasons several articles have reported measurements of the anterior loop length, diameter of incisive canal, incidence of lingual foramen, incisive canal, and mental foramen.

Preoperative radiographic examination is an essential diagnostic method to determine these anatomical structures. Cross-sectional imaging, including different kinds of tomographies and magnetic resonance, are the radiographic methods of choice for preoperative evaluation and recommended to assist preoperative examination (10-12). Tomographic techniques provide adequate information concerning the overall shape of the interforaminal region of the mandible. In addition the optimum image quality, the excellent geometric accuracy and the low radiation dose, together with the ease of handling make cone-beam computed tomography (CBCT) a suitable system for implant treatment planning of the anterior mandibular area (13-15).

The objective of this study was to measure the anatomical landmarks using CBCT in the interforaminal region to determine the safety of drilling in this area.

## Material and Methods

**Figure 1:**
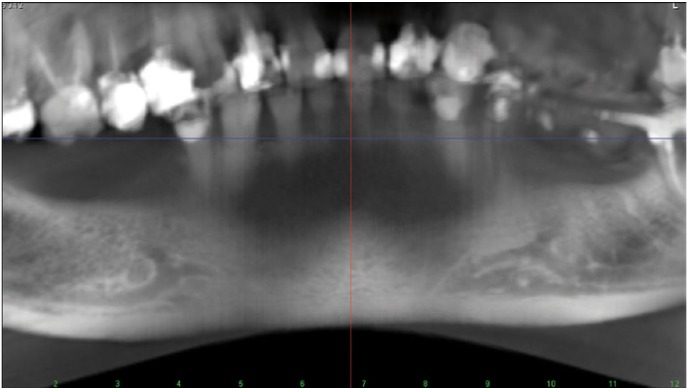
The panoramic view of anterior loop and incisive canal.

**Figure 2:**
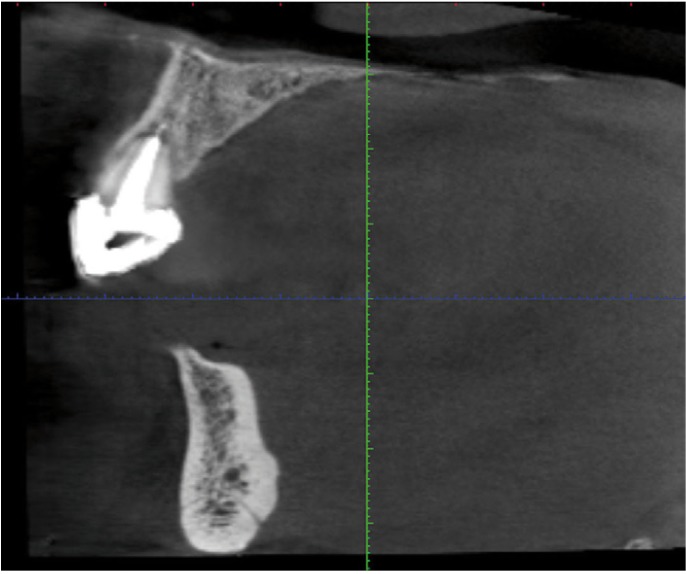
Reformatted cross-sectional image of incisive canal.

This experimental study included 96 CBCT scans from patients with partially edentulous mandibles .The selected scans had paraxial slices with a slice thickness of 1 mm slices .The scans had been taken as part of a diagnostic procedure for implant supported rehabilitation. The age of the patients ranged from 20 to 77 years (mean age: 46.60 years), 48% of which were females and 52% were males. 68% of the subjects were 35-58 years old. All of the mandibles under investigation were partially edentulous in the anterior or posterior regions. The interforaminal region was examined in the CBCT scans. The CBCT examinations were obtained using the Promax 3D (Planmeca, Finland) CBCT unit, which automatically sets the appropriate exposure parameters for each patient. The acquired images were processed with the Romexis software. In this study, contiguous cross-sectional images with a 1-mm step were used. Measurements were done using the measurement tools of the Romexis software under magnification ×3 by a Samsung monitor (SyncMaster 2243BWX, Resolution 1680 ×1050 and Brightness 300 cd/m2). All the measurements were carried out by one of the authors, experienced in CT scan interpretation and implant dentistry. In order to standardize the procedure, the reformatted images between mesial border of right and left mental foramen were selected from the panoramic, as this area corresponds to the interforaminal region. Axial panoramic and reformatted cross-sectional images were carefully evaluated to study important anatomical landmarks in the interforaminal region included:

1. The visibility of the mental foramen, anterior looping, incisive canal and lingual foramen, were scored using a four point rating scale (good, moderate, poor and no visibility).

2. Diameter of incisive canal was measured in four points with 2 mm distance from each other (Fig. 1).

3. Mesiodistal length of anterior loop (Fig. 1).

4. Distance of incisive canal from lower, lingual and buccal border of the mandible on reformatted cross-sectional images, was taken 2 mm apart from each other (Fig. 2).

5. Distance of mesial border of right and left mental foramens were measured by subtracting the slice number of the mesial wall of the right mental foramen from the slice number of the mesial wall of the left mental foramen. The measurements from all images on interforaminal region were recorded on a chart. These measurements were repeated twice on separate days by the same examiner without knowing the recorded data of previous days, and the coordinates were averaged. Weighted Cohen’s Kappa used for the intraobserver visibility reliability and overall score of 0.93 was obtained. Descriptive statistical tests were used to analyze the data with SPSS/win 15 Software. In addition to descriptive statistical tests, the effect of gender and age on the visibility of anatomic landmarks described was investigated using Chi-squere and independent samples t-test, respectively. A 5% level of significance was chosen.

## Results

-Visibility rating of anatomical landmarks

**Figure 3:**
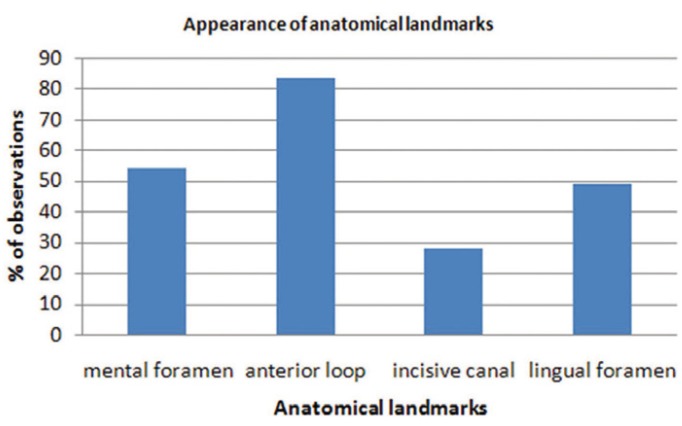
Results of the frequency distribution of anatomical landmarks on CBCT images.

**Figure 4:**
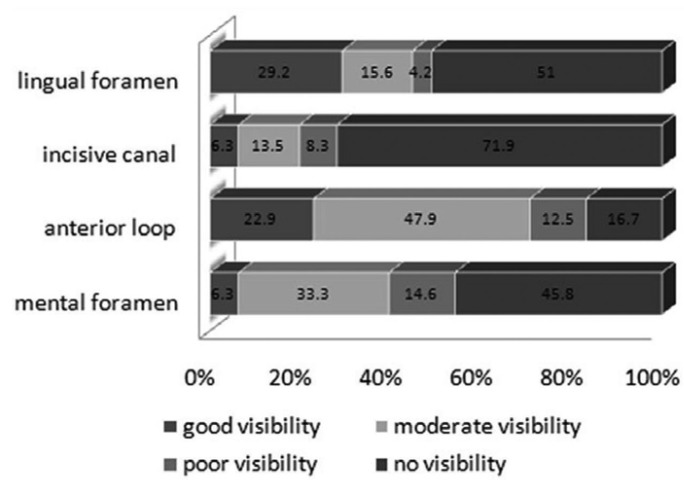
Results of the visibility rating of the anatomical landmarks on CBCT images.

The appearance and visibility of the anatomical landmarks on the images is shown in (Figs. 3,4).

Right mental foramen was detected in 100% of the cases. However, only 68.8% of the cases had good visibility. Left mental foramen was also observed in 100% of the cases among which 67.7% had good visibility. The incisive canal could be detected in 71.9% of the cases in the inter-foraminal region with good visibility in 39.6% of the cases. Anterior looping could be visualized in 83.3% of the cases in the right and in 62.5% of the cases in the left side. Finally, the appearance of lingual foramen is 49.0%.

-Mental foramen

The mean size of anterior loop was 3.54 mm with SD=1.41(right: mean=3.6 mm, SD=1.0 and left: mean=3.2mm, SD= 1.5). The mean distance of right and left mental foramen from midline were 25.9mm (SD=4.8) and 24.9mm (SD=4.7), respectively. Therefore, the mean inter-foraminal distance was 50.8mm. The distance of lingual border of mental foramen was 5.05mm (SD=2.28) from the lingual border of mandible (right: mean=5.2 mm, SD=3.9 and left: mean=4.8mm, SD= 1.5).

-Incisive canal

Mean diameters of right and left incisive canal were 1.49mm (SD=0.70) and 1.44mm (SD= 0.48), respectively. Total mean obtained was 1.47mm (SD=0.50). Diameter of incisive canal from its origin to midline, measured in four points was gradually decreasing in both sides. Mean distance of incisive canal from lingual border of the mandible was 4.46mm (SD=1.40) (right: mean=4.58mm, SD=1.51 and left: mean=4.36mm, SD= 1.51). Mean distance of incisive canal from buccal border of the mandible was 3.48mm (SD=1.17) (right: mean=3.40mm, SD=1.26 and left: mean=3.48mm, SD= 1.25). Mean distance of incisive canal from lower border of the mandible was 8.72mm (SD=1.43) (right: mean=8.98mm, SD=1.68 and left: mean=8.64mm, SD= 1.51).

-Lingual foramen 

**Figure 5:**
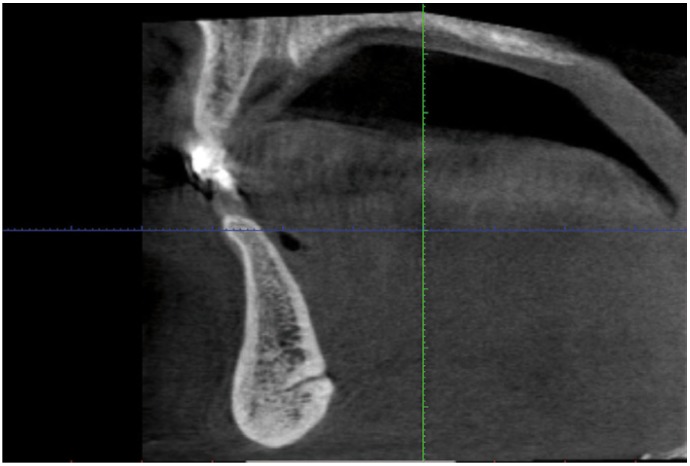
Reformatted cross-sectional image of lingual foramen canal.

The lingual foramen was seen as a canal in reformatted cross sectional images (Fig. 5). The mean width of lingual foramen canal was 0.8mm (SE=0.09). The mean distance of lingual foramen from the lower border of the mandible was 8.85mm (SD=2.99).

-Appearance of anatomical landmarks in relation to age and gender 

Chi-squere statistics revealed no significant effect of gender on the visibility of the anatomical landmarks. Independent sample t-test did not demonstrate any deference of incisive canal distance from mandibular borders in both genders. There was no correlation between age and diameter of incisive canal according to Pearson regression test (R=0.079).

## Discussion

Meticulous care taken in the diagnosis and the treatment planning for the patient critically determine the long term prognosis of an implant restoration. Many diagnostic techniques have been developed to aid the clinician in the pre-surgical planning of dental implants one of which is pre-operative radiographic examination. This method is an essential diagnostic method to determine the size, location and angulation of each dental implant according to the anatomical landmark. The mental foramen is an important landmark where implant placing is to carry out in the foraminal region of the mandibular arch. To avoid the mental nerve injury during implant surgery, it is necessary to define the location and also the possibility of being an anterior loop of the mental nerve mesial to the mental foramen. Sensory dysfunction due to nerve damage in the foraminal area can occur if the inferior alveolar or mental nerve is damaged during osteotomy. Since improper placement of implant in the intermental area causes pain and discomfort, reported in several cases, a zone of safety (in millimeter) for implant placement must be determined. The present study describes the good visualization and 100 % appearance of mental foramen on CBCT scans of mandible. These data are in good agreement with other observational studies such as Jacobs et al. that found 100% appearance of mental foramen with good visibility (16). In this study the mean distance of the mental foramen from midline was 25mm. Agthong et al. and Neiva et al. indicated that the foramen was 28 mm and 27.6 mm distally from the midline, respectively (17,18).

The anterior loop, one of the anatomical landmarks, is obviously seen in this research. It has been previously proved that the anterior loop may change frequently with anatomical variations bearing a lumen of approximately 2 mm (19). The present study shows the appearance of anterior loop on the CBCT scans. The anterior loop was generally seen in 84.4 % of total cases on the recorded CBCT scans of mandible, while 74% of observed loops have great to moderate visibility. The mean length was 6 ± 1.5 mm. Jacobs et al. (16) found an anterior loop of the mental nerve in 7% of CT scans of patients. In the Rosenquist study which performed dissection of the ramification of the inferior alveolar neurovascular bundle unilaterally, anterior loop was found in 74.13% of patients and the length of which ranged from 0 to 1 mm (the mean was 0.15) (8).

The possibility that the loop of the anterior loop may not exist does not necessarily mean that implants can be safely placed close to the mental foramen. The sensory disturbances of the lower lip have been reported due to the direct trauma to the anterior looping of the mental nerve (20).

Another landmark studied in this research is the incisive canal. This is a continuation of the mandibular canal anterior to the mental foramen. The present study shows that the incisive canal is appeared in 83.3 % of all cases, while the appearance of the incisive canal on CBCT of mandible has moderate or good visibility in 70.8% of total cases. The average diameter was defined 1.47±0.50 inferior to the tooth apex. According to Mardinger, the canal was found in 56% cases and the diameter ranged from 0.48mm to 2.9 mm (21). Jacobs et al. identified the incisive canal in 93% of the spiral CT scans of the lower jaw (16). Bavitz et al. performed measurements on cadaver mandibles found a mean diameter of 1.3mm with a range of 0.5 to 2 mm which can be compared with the present study (22). Polland et al. studied the incisive canal in edentulous cadaver mandibles and found an ill-defined incisive canal (23). The canal contains the incisive bundle that innervates the teeth in the anterior segment. Its precise anatomy and intermedullary content is important during implant insertion. One possible result from traumatizing the neurovascular bundle is sensory disturbance. Kohavi and Bar-Ziv describe a case where pain and discomfort resulted from implants placed in the intermental area. CT images revealed that the implants were placed through a large lumen of the incisive canal (20,22). Rosenquist found that the incisive bundle caused implant failure, by migration of soft tissue around the implant, thus preventing osseointegration (8). Wismeijer et al. studied 110 edentulous patients who were treated by overdenture supported by two or four implants. Although the mental foramen was always identified during the operation and all implants inserted at least 3 mm medial to the anterior border of the mental foramen, permanent sensory disturbance in the lower lip was noted in 7% of the cases. Several explanations may be available such as direct trauma to the incisive neurovascular bundle (9). Another factor might also be related to indirect trauma to the incisive canal bundle, causing hematoma in the closed chamber that may produce pressure on the mental nerve. Thus the canal could play an important role in successful osseointegration and prevention of postoperative sensory disturbances.

The present study shows the appearance of lingual foramen in 49 % of all CBCT scans of mandible. Figure 4 shows the distribution of the visibility quality of the observed lingual foramen. 44.8 % of total cases has moderate to good visibility. The lingual foramen was seen as a canal in the reformatted cross sectional images with mean width of 0.8 mm. The lingual foramen is situated in the midline, leveled with or superior to the genial tubercles. A pilot study by Mc Donnel revealed an incidence of 49% of the lingual foramen on periapical radiographs of the mandibular incisor region in adult population (4). The study suggested that the lingual foramen was a consistent finding to the lingual side of the mandible in the midline, being present in over 99% of the dried specimen examined. The foramen was definitely visible in 81% and 82% in Makris based on CBCT and Jacobs based on CT studies, respectively (14). The lingual artery has sufficient size to present difficulty in control of hemorrhage intraosseously or in soft tissue. Also in view of its position it could be a factor in implant placement in the midline (4).

Overall, in all observed landmarks it should be mentioned that the thickness of the CBCT images is 1mm, limiting the accuracy of the measurements. Also the experience and skill of the observer is important during pre-operative planning phase. The high detection rate of the landmarks in the anterior region of the mandible using CBCT indicates the potentional high preoperative value of CBCT for surgical procedures in the anterior mandible.

## Conclusions

It can be concluded that there may be large variations in the anatomical landmarks in the foraminal region; one should not assume that a fixed distance mesially from the mental foramen will be safe. With the increased interest in performing thorough pre-operative planning prior to oral implant surgery in interforaminal region, cross-sectional images may be considered for obtaining more information on the anatomical landmarks. Tomographic techniques provide adequate information concerning the overall shape of the interforaminal region of the mandible revealing any anatomical landmarks which may be of some value for preoperative planning. Intra-operative examination of the vital structures is indispensable for the safe installation of implants in the interforaminal area of the mandible. Also the diameter of the canals and foramens should be investigated on a case-by-case basis to determine the appropriate location for each individual. Also the diameter of the canals and foramens should be investigated on a case-by-case basis to determine the appropriate location for each individual.
